# Assessing the diagnostic performance of a novel RT-PCR fluorescence method for the detection of human plasmodium species

**DOI:** 10.1371/journal.pone.0272094

**Published:** 2022-08-04

**Authors:** Melkamu Bedimo Beyene, Seifegebriel Teshome, Yehenew A., Zewdu Terefework, Antoine R. Stuitje, Tamrat Abebe, Habtamu B. Beyene

**Affiliations:** 1 Department of Public Health, College of Health Sciences, Bahir Dar University, Bahir Dar, Ethiopia; 2 Department of Microbiology, Immunology and Parasitology, Addis Ababa University, Addis Ababa, Ethiopia; 3 MRC-ET Molecular Diagnostics PLC, Addis Ababa, Ethiopia; 4 MRC-Holland, Amsterdam, The Netherlands; Kyung Hee University, REPUBLIC OF KOREA

## Abstract

**Background:**

Malaria elimination effort is hampered not only by the lack of effective medication but also due to the lack of sensitive diagnostic tools to detect infections with low levels of parasitemia. Therefore, more sensitive and specific high-throughput molecular diagnostic approaches are needed for accurate malaria diagnosis.

**Methods:**

In the present study, the performance of a novel single-tube MC004 real-time polymerase chain reaction (PCR) assay (MRC-Holland, Amsterdam, the Netherlands) was assessed for the detection of infection and discrimination of *Plasmodium species*. Blood samples (n = 150) were collected from malaria suspected patients at Adama malaria diagnosis and treatment centre, Adama, central Ethiopia. The positive predictive value (PPV), negative predictive value (NPV), analytical sensitivity and specificity of the assay were assessed against the conventional microscopic method.

**Results:**

Plasmodium species were detected in 59 (39.3%) of the samples by microscopy and in 62 (41.3%) by the novel MC004 RT-PCR. *Plasmodium vivax*, *Plasmodium falciparum* and mixed infections with *Plasmodium falciparum* & *Plasmodium vivax* accounted for 47.5%, 40.6% and 11.9% respectively as detected by microscopy. The MC004 RT-PCR assay identified 59.7% and 40.3% of the samples positive for *Plasmodium vivax* and *Plasmodium falciparum* respectively. The sensitivity, specificity, PPV, and NPV of the MC004 RT-PCR assay were 95.8%, 97.8%, 92%, and 98.9%, respectively. No mixed infections were detected using the MC004 assay.

**Conclusion:**

The MC004 RT-PCR assay is a useful tool for the early detection of malaria and identification of *Plasmodium* species with a high degree of sensitivity and specificity. Due to its high sensitivity, and simplicity (being a single-tube assay), the MC004 is suitable for use in clinical settings and epidemiological studies.

## Introduction

Malaria remains one of the major public health problems particularly in the tropical and subtropical regions [1]. In 2020, approximately 214 million malaria cases and 627,000 deaths were recorded globally. The prevalence of malaria is highest in Africa and South East Asia. In Africa alone, an estimated 228 million cases were documented in 2020 contributing to about 95% of the cases worldwide [2]. Malaria in humans is exclusively caused by infection with one of the five distinct *Plasmodium* species (*P*. *falciparum*, *P*. *vivax*, *P*. *ovale*, *P*. *malariae* or *P*. *knowlesi*). In Africa, *Plasmodium falciparum* is the most common cause of malaria while, *Plasmodium vivax* is the most widely distributed species outside Africa [3, 4].

Ethiopia is one of malaria endemic Sub-Saharan African countries known for its diverse climatic conditions. The complex interaction between different climatic conditions including variable winds, seasonal rains, and ambient temperature for mosquito breeding creates diverse micro-climates that result in a seasonal and dynamic malaria transmission [5]. Despite the decreasing trend in the overall burden observed in the last few years, malaria still remains one of the major challenges particularly in the rural areas. Roughly, 52 million people (68%) of the population in Ethiopia live in malaria endemic areas that make up 75% of the total land mass [6]. The two predominant plasmodium species commonly found in Ethiopia are *Plasmodium falciparum* and *Plasmodium vivax*. *P*. *falciparum* is responsible for 60 to 70% % of the total reported malaria cases [7, 8]. However, in some areas with an altitude below 2,000 meters above sea level, *P*. *falciparum* accounts for up to 77% of malaria cases [5].

Effective diagnostic tools are required for a reliable detection of infection and identification of plasmodium species to improve patient care and management. Molecular techniques such as PCR are now becoming increasingly popular throughout the world [9]. There are many types of molecular diagnostic techniques that have been developed for the detection of malarial infections in humans. Some of the techniques are based on the amplification of plasmodium DNA followed by the real time quantitative PCR (RT-PCR) assay [10–12] or qualitative detection of plasmodium species with high analytical sensitivity and specificity [13, 14].

Compared to the molecular diagnostic approaches targeting the plasmodium DNA, microscopy and rapid diagnostic tests (RDT) display enormous limitations. Both microscopy and RDT are not suitable for parasite load determination, accurate species identification and detection of drug resistant parasite strains. Previous studies have demonstrated higher sensitivity and specificity of RT-PCR based techniques compared to microscopic and RDT for the diagnosis of malaria. Of note, among malaria patients and immigrants from different endemic countries [15, 16], a PCR showed a significantly higher analytical sensitivity and specificity to detect human plasmodia in blood samples. Moreover, unlike conventional microscopic method in which only 30 parasites were detected per μL of blood, RT-PCR detected as few as 4 parasites per μL [16].

The main aim of the present study was to assess the performance of a novel MC004 RT-PCR assay (MRC-Holland, Amsterdam, the Netherlands) against light microscopy to the detection and identification of human plasmodium species. The MC004 RT-PCR technique is a single-tube assay that has been developed for the simultaneous detection and identification of human plasmodia including *P*. *falciparum*, *P*. *vivax*, *P*. *malariae*, and *P*. *knowlesi*. The initial evaluation study using reference samples, patient samples and synthetic controls has been recently published [17]. We build upon this to further confirm the assay’s performance in a different population setup. It is known that the performance of diagnostic tests are affected by the prevalence and positive predictive value of a given disease. In the current paper, a different set of participants with different characteristics and malaria parasite carriage were included compared to the patients enrolled in our earlier publication.

## Methods and materials

### Study area and population

A prospective cross-sectional study was conducted on blood samples collected from individuals who visited Adama malaria diagnosis and treatment centre, central Ethiopia between September 2017 to December 2017. The centre is one of the few malaria diagnostic and treatment facilities in the country located in South East of the capital city, Addis Ababa. Adama is one of the well-known malarious cities in the country due to its low altitude (< 2000 m above sea level) and other factors that favour mosquito breeding [18].

### Sample collection and preparation

Individuals who were suspected of malaria were recruited. Accordingly, 150 participants who visited the diagnostic centre were recruited on a convenience basis. Patients who were taking anti-malarial drugs during the recruitment period or within two weeks before the start of data collection as well as those participants who were critically ill were excluded from the study. All consenting individuals who are not taking anti-malaria drugs were enrolled. Five (5) millilitres of blood was collected from each subject and transferred into a sterile EDTA tube. Blood films (both thin and thick) were prepared for each sample during collection. A blood spot was also collected on Whatman filter paper from each participant.

### Microscopic examination

Thick and thin blood films (10% Giemsa stained smears) were examined under microscope to detect and identify plasmodium species. The blood slides were examined at x100 magnification power with an oil immersion by the certified Medical Laboratory Scientists (at least two independent readers involved). The investigator was blinded of the first reader. Blood films were reported ‘negative’ if no parasite/s were observed after examining 300 microscopic fields.

### DNA extraction, amplification and detection

DNA was extracted from the EDTA preserved whole blood samples followed by isolation using the QIAamp DNA Blood Mini QIAcube Kit on a QIAcube instrument (Qiagen, Hilden, Germany). The DNA extract was subsequently eluted with 100 μL of elution buffer. A 25 μL reaction mix containing 23 μL of MC004 master mix (MRC-Holland, Amsterdam, the Netherlands) and 2 μL of template was used in each run. The reaction mix essentially contains the forward primers (1.25 μM), reverse primers (0.25 μM), probes (0.5 μM), fluorescent compound, dNTPs, reaction buffer, and Taq DNA polymerase. DNA amplification was achieved by setting the reaction condition as follows: 95°C for 3 min, then 50 cycles of 95°C for 15 s, 60°C for 30 s and 68°C for 40 s. Temperature was increased from 25 to 69.4°C (at 0.4°C per 5 s) for melting curve analysis.

### MC004 RT-PCR assay

Microscopically confirmed cases (including slide positive and negatives samples) were further analysed by MC004 RT-PCR assay. The primers and probes are designed for the highly conserved mitochondrial Cytochrome b gene of all five human *Plasmodium species*. Primers used for primary PCR reaction on G-block dilutions are TCGCTTCTAACGGTGAAC (Forward) and ACCGCTAGTGTTTGCTT (Reverse). The MC004 assay is based on multiplex nucleic acid amplification. It is designed to detect mitochondrial DNA encompassing the cyclo-oxygenase 3 (COX-3), cyclo-oxygenase 1 (COX-1) and cytochrome b (CYTB) genes having different melting temperatures. The detailed methodology has been described in detail elsewhere [17].

*Plasmodium* species identification was based on the differential melting temperatures of the respective plasmodial target sequences. The probes designed to distinguish between all 5 species of malaria that are identical to the species sequences. As controls, melting curve PCR products on ±50 molecules of the corresponding CytB gene-blocks of the five human plasmodial species were generated on BIO-RAD CFX 96. Quality control samples encompassing the positive control samples for each plasmodium species are provided with the MC004 (by MRC Holland). In situations where no amplification curve is observed the test result is inferred invalid. These situations were not evident in the present study.

### Data analysis

Data were entered into Microsoft Excel (Microsoft Office 365). The MC004 RT-PCR assay test performances such as sensitivity and Cohen Kappa test for agreement between the MC004 RT-PCR and microscopic method were determined using the free version of MedCalc statistical software. SPSS Software statistical package version 20.0 or R version 3.6.2 was used to analyse the socioeconomic data, and to compute frequencies and percentages of microscopic and PCR results.

### Ethical considerations

Ethical clearance was obtained from Addis Ababa University, College of Health Sciences Institutional review board (IRB) (protocol number 017/17/DMIP). The socio-demographic data and blood sample were collected after informed consent was obtained from each participant.

## Results

### Characteristics of participants

A total of 150 participants were recruited in this study of which 119 (79.3%) were males. The mean (SD) age of the study participants was 33±14 years. The age of participants range from 16 to 75 years. Out of the 150 participants, 64.7% were under the age of 33. Some 82.7% of the study participants reported to have had malaria in the past (Table 1).

**Table 1:** Characteristics of the study participants.

Characteristics	n	Percent
**Total participants**	150	100
**Sex**		
Men	119	79.3
Women	31	20.7
**Age group (years)**		
15–25	61	40.7
26–35	42	28.0
36–45	20	13.3
> = 46	27	18.0
**History of malaria**		
Yes	124	82.7
No	26	17.3
**Malaria positivity rate**		
**Microscopy**		
Positive	59	39.3
Negative	91	60.7
**Parasite stages**		
Ring stage	28	47.5
All parasite stages	31	52.5
**MC004 Real time PCR**		
Positive	62	41.3
Negative	88	58.7

### Microscopy

**Fig 1:**
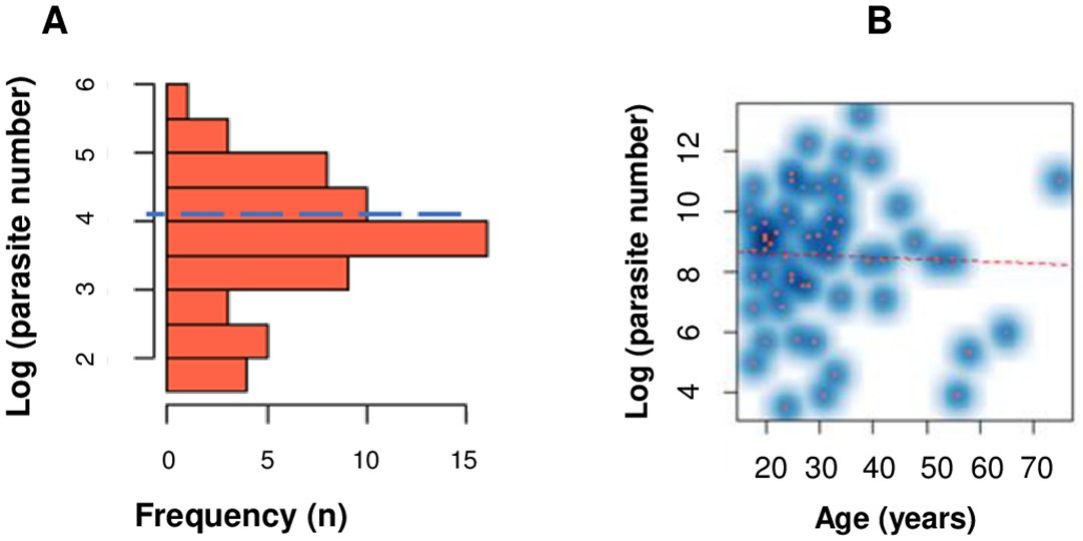
Parasitaemia level using microscopic examination. (A) Smooth density scatterplot to show the distribution. (B) Age versus parasitemia.

A total of 59 (39.3%) of all samples were tested positive by microscopy. Among the malaria positive individuals, 51(86.4%) were males. *Plasmodium vivax and Plasmodium falciparum* accounted for 28 (47.5%) and 24 (40.6%) of all positive cases respectively. Mixed infections with *P*. *vivax* and *P*. *falciparum* were identified in 7 (11.9%) of samples. The predominant parasite developmental stages observed under the microscope were the mix of ring stage (trophozoite), gametocyte and schizont stage (observed in 31 (52.5%) of the samples). Ring stage alone was reported in 28 (47.5%) of the positive slides. The mean (SD) log10 parasite count was 4.05 (4.9) as shown by the blue line in the histogram below (Fig 1A). We plotted a smooth density scatterplot to show the high parasitaemia load in younger age (Fig 1B).

### Performances of MC004 real-time PCR assay

Some 62 (41.3%) of the examined samples were positive and 58.3% were negative for plasmodium species using the novel MC004 RT-PCR method. Of the positive cases, 37 (59.7%) and 25 (40.3%) were *P*. *vivax* and *P*. *falciparum* species respectively. No mixed infection was identified using MC004 RT-PCR. Seven participants were identified as having a mixed infection by microscopy from which six were tested as *Plasmodium vivax* and the other one was tested as *P*. *falciparum* by the MC004 RT-PCR. From the 59 malaria positive samples detected by microscopy, 47 (79.7%) and from 62 MC004 RT- PCR positive participants, 49 (79%) had reported previous history of malaria. There was no a statistically significant association between the previous history of malaria infection with the malaria positivity rate using both methods.

A total of 59 (39.3%) of samples were tested positive by Microscopy and novel RT-PCR while 88 (58.7%) tested negative using both microscopy and novel RT-PCR. Only 3 (2%) of the samples were tested positive by RT-PCR but tested negative by microscopy. No sample/s were found positive using microscopy but negative by novel RT-PCR. There was no a statistically significant difference between the novel RT-PCR and the microscopic method at alpha <0.05.

### Comparison of MC004 real-time PCR assay with microscopy as a gold standard

Out of the 62 participants that were tested positive by novel MC004 RT-PCR, 59 (95.2%) were positive by microscopy whereas the 88 samples that were tested negative by novel MC004 RT-PCR were also negative by microscopy. Only three smear-negative subjects were found positive by novel MC004 RT-PCR. The sensitivity, specificity, PPV, and NPV of the MC004 RT-PCR were 100%, 96.7%, 95.2%, and 100% respectively. The positive and negative likelihood ratio were also calculated and it was 25 and 0 respectively. The percent agreement between the two tests were 98% and the kappa value was 0.958 which is an excellent agreement for the identification of the plasmodium parasites (Table 2).

**Table 2:** Agreement between microscopy and MC004 RT-PCR for mono parasite diagnosis.

		**Microscopy**			**Cohen’s kappa test**
		**Positive**	**Negative**	**Total**	
**MC004 RT-PCR**	Positive	59	3	62	95.8%
	Negative	0	88	88	
Total		59	91	150	

From 24 *Plasmodium falciparum* identified by microscopy, 23 (95.8%) were also positive for same species using MC004 RT-PCR and one sample was tested as *P*. *vivax*. Two samples were tested positive by MC004 RT-PCR but these samples were tested as negative and mixed infection with both *P*. *vivax* and *P*. *falciparum* by microscopy. Of the 3 samples tested negative by the microscope, two were *P*. *vivax* and one was *P*. *falciparum*. The sensitivity, specificity, PPV, and NPV of the MC004 RT-PCR were 95.8%, 97.8%, 92%, and 98.9%, respectively. The positive and negative likelihood ratio of the methods were 43.4 and 0.04 respectively. The percent agreement between the two tests were 97.4% and the kappa value was 96.5%.

All the 28 *Plasmodium vivax* species identified by microscopy were also tested positive for *Plasmodium vivax* using MC004 RT-PCR. But there were 9 additional *Plasmodium vivax* species that were identified by the MC004 RT-PCR of which six were reported to be mixed infection, one *P*. *falciparum* and the other two were negative by microscopy. The sensitivity, specificity, PPV, and NPV of the MC004 RT-PCR were 100%, 90.7%, 75.7%, and 100%, respectively. The positive and negative likelihood ratio of the methods were 10.8 and 0 respectively. The percent agreement between the two tests were 77.3% and the kappa value was 61% which is a good agreement between the two tests in identifying *Plasmodium Vivax* species.

In terms of identifying the *Plasmodium* species, the MC004 RT-PCR-based assay showed a reasonable agreement with microscopy (kappa value = 87.2%). This agreement indicates the ability of the MC004 RT-PCR to identify malaria species was agreeable with microscopy except the limitations in identifying the mixed infection (Table 3).

**Table 3:** Identification of *Plasmodium* species by using microscopy and RT PCR.

		**Microscopy**				**Total**
		***P*. *falciparum***	***P*. *vivax***	**Mixed**	**Negative**	
**MC004 RT-PCR**	*P*. *falciparum*	23	0	1	1	25
	*P*. *vivax*	1	28	6	2	37
	Negative	0	0	0	88	88
Total		24	28	7	91	150

## Discussion

Accurate diagnosis of malaria is of paramount importance for proper treatment and management of patients with febrile illnesses [8]. Moreover, the identification the *Plasmodium* species is not only important for establishing a successful treatment regimen, but also for designing effective malaria control measures in endemic regions. Misidentification of the *Plasmodium* species could cause a serious public health concern as it has implications to parasite clearance times, recrudescence and drug resistance [19–21]. Today, the use of highly sensitive and specific techniques such as PCR are becoming increasingly popular for malaria diagnosis including in resource limited settings [22–25]. Increased access to molecular diagnostics leads to overall improvement of malaria diagnosis, treatment and prevention of malaria [26]. Given the inherent variability in many PCR protocols some assays are suitable for point of care (POC) applications [27, 28] while others are meant primarily to detect low parasitemia [29, 30]. Consequently, regular evaluation of the analytical performance of diagnostic tests will help choose the right tool for the right setting and or purpose.

The present study was therefore, designed to assess the diagnostic performance of a novel MC004 RT-PCR assay against direct microscopy as the ‘gold standard’. The MC004 assay specifically targets Plasmodium mitochondrial DNA for amplification, and includes post-PCR melting curve analysis based on three different molecular beacon probes. More details on the methodology can be found in our previous study [17]. The MC004 RT-PCR assay for malarial parasites were in almost perfect agreement with microscopy (k = 0.958, specificity = 96.7% and a sensitivity of 100%. This was different from results reported by Gatti *et al*. (kappa value = of 0.89, and a specificity of 98.2% and a sensitivity slightly lower (91.0%) [31]. Our findings also show difference with Kattenberg JH *et al*. who reported a specificity of 65.7% and a sensitivity of 97.3% [16]. The observed differences might be explained by the inherent difference in diagnostic tools, the prevalence of malaria in the study settings and possibly other factors. It is also important to note that, microscopic examination is dependent on the skill/experience of the microscopist.

The positivity of *P*. *falciparum* and *P*. *vivax* using MC004 RT-PCR were 40.3% and 59.7% respectively which is comparable with other studies that have been conducted in Ethiopia [32–34]. In the similar study site, previous studies reported prevalence of 61–71% for *P*. *vivax* and 26–39% for *P*. *falciparum* [35, 36]. In contrary to our study, studies conducted in many regions of the country reported a slightly different finding. In a study conducted by Meresa *et al*, *Plasmodium falciparum* infection accounted for 58.2% while *P*. *vivax* was 35.5% [37]. Similarly, others reported *Plasmodium falciparum* as a predominant species [38–40]. These findings highlight that there is a heterogeneity in the distribution of malaria parasites which is presumably governed by the diverse demographic and climatic conditions across the country.

In the current study, the agreement between microscopy and the MC004 RT-PCR in identifying plasmodium species was 87.2% which goes in line with the findings from studies conducted in Thailand by Coleman *et al*. and in Myanmar by Kang *et al* [41, 42]. These studies reported that there was 87% and 87.3% agreement between PCR and microscopy respectively. In the present study, the MC004 RT-PCR correctly identified 95.8% of microscopy identified *Plasmodium falciparum* samples and 100% of microscopy identified *P*. *vivax*. This was also similar with the resulted reported by Coleman et al who demonstrated a 96% and 100% *P*.*falciparum* and *P*.*vivax* identification by PCR and microscopy [41, 42]. The diagnostic accuracy of the molecular technique in detecting *P*. *falciparum* infections was almost perfect, with a sensitivity of 95.8% and a specificity of 97.8%. This was comparable with Gatti *et al*. who demonstrated a sensitivity of 98.9% and a specificity of 100% [31]. Patsoula *et al*. also reported perfect agreement (i.e 100% sensitivity and specificity) between the PCR and the microscopy in the identification of *P*. *falciparum* species [43].

With regards to detecting the *P*. *vivax* by the MC004 RT-PCR compared to microscopy, we have found a lower agreement (Kappa = 61%) although we observed a100% sensitivity and 90.7% specificity. The main reason for this lower agreement is that the MC004 RT-PCR based assay couldn’t trace out the mixed infections. Instead, it reports them as a mono parasite infection. The sensitivity of this test was similar with Patsoula *et al*. that reported 100% sensitivity and 100% specificity. In this study, no mixed infection was reported [43]. In terms of the mixed infections that were identified by the microscopy, the MC004 RT-PCR assay showed poor concordance with the ‘gold standard’. Such poor performance in the identification of mixed infections has been described by Gatti *et et al* [31]. But it has been suggested that, PCR is more sensitive and reliable than microscopic examination in differentiating mixed infections [42]. The variation seen in these studies for the detection of malarial infection could be due to methodological differences in the various study protocols.

The present study had limitations to mention. The cross-sectional study design and a relatively small sample size did not allow for the examination of temporal relationship between malaria and other variables. Moreover, the detection of rare parasites particularly in mixed infections may be limited. Another methodological related limitation was the lack of other PCR protocols to compare our recent test method with.

## Conclusions

In conclusion, the present MC004 RT-PCR assay is a highly sensitive and specific method for the detection of malarial parasites, especially *P*. *falciparum* and *P*. *vivax*. Given the higher sensitivity and specificity of this novel technique, it has a great role in the diagnosis of malaria and identification of *plasmodium* species. This will in turn improve patient care and management as well as effective monitoring of control and elimination programs.

## Supporting information

S1 Data (CSV)
